# COVID-19 infection and BNT162b2 vaccine triggering sarcoid-like lesions in the same patient. Response to: Sarcoid-like reaction in a patient recovering from COVID-19 pneumonia

**DOI:** 10.1016/j.jdcr.2021.12.042

**Published:** 2022-01-21

**Authors:** Teresa Grieco, Alfredo Rossi, Patrizia Maddalena, Alvise Sernicola, Luca Ambrosio, Pasquale Fino, Vito Gomes

**Affiliations:** a Dermatology Unit, Department of Clinical Internal Anesthesiological Cardiovascular Sciences, “Sapienza” University of Rome, Italy; b Pathological Anatomy Unit, Ospedale San Filippo Neri, Rome, Italy

*To the Editor:* Behbahani et al^1^ recently reported in your journal a rare case of sarcoid granulomas induced by cross-reactivity to SARS-CoV-2 infection. Since then, different types of vaccines against COVID-19 have been produced. Among these, BNT162b2 (Comirnaty, Pfizer/BioNTech) is a messenger RNA vaccine encoding the receptor-binding domain (RBD) of the SARS-CoV-2 spike protein.^2^ We report a case of cutaneous sarcoid-like granulomatous reaction in a patient with COVID-19, reactivated by the administration of BNT162b2 vaccine.

A 73-year-old Caucasian woman presented in July 2021, complaining of painful skin lesions on her arms and legs.

Physical examination revealed erythematous-violaceous nodules that were painful and distributed bilaterally and asymmetrically on her arms and legs, suggesting erythema nodosum-like lesions (Fig 1). On palpation, these nodules were mobile, warm, and tender; however, there was no appreciable lymphadenopathy. No significant comorbidities were present, and past medical history was unremarkable, except for a mild SARS-CoV-2 infection in January 2021, treated with acetaminophen. The patient denied being on any medication. Erythema nodosum-like skin lesions were first reported in March 2021 and underwent spontaneous regression in the following weeks but recurred in June 2021, 15 days after administration of the first dose of BNT162b2 vaccine. On ultrasound examination performed in July 2021, the skin nodules appeared as multiple dermo-hypodermal inflammatory lesions with a nonhomogeneous hypoechoic pattern. Skin biopsy revealed noncaseating giant-cell sarcoid granulomas surrounded by few lymphocytes in the papillary and reticular dermis and dermo-hypodermal junction (Fig 2). Periodic acid–Schiff, Ziehl-Neelsen, and Giemsa stains were negative. Immunohistochemistry showed a prominent CD4^+^ T-lymphocyte infiltrate, with an overall high CD4/CD8 ratio. Angiotensin-converting enzyme and calcium levels were within the normal range, and C-reactive protein was 1.79 mg/dL. Chest X-ray, electrocardiography, and eye examination were unremarkable. A diagnosis of SARS-CoV-2-induced sarcoid-like granulomatous reaction was made. We started methylprednisolone 16 mg twice daily for 1 month, slowly tapering the dose over the following month. The persistence of painful lesions prompted us to initiate steroid-sparing therapy with cyclosporine 5 mg/kg daily. The patient was reassessed after 4 weeks of treatment, showing a stationary cutaneous clinical picture with reduction of associated pain. Considering the symptomatic response achieved, the patient is currently continuing treatment and is scheduled for follow up after 1 additional month.

**Fig 1:**
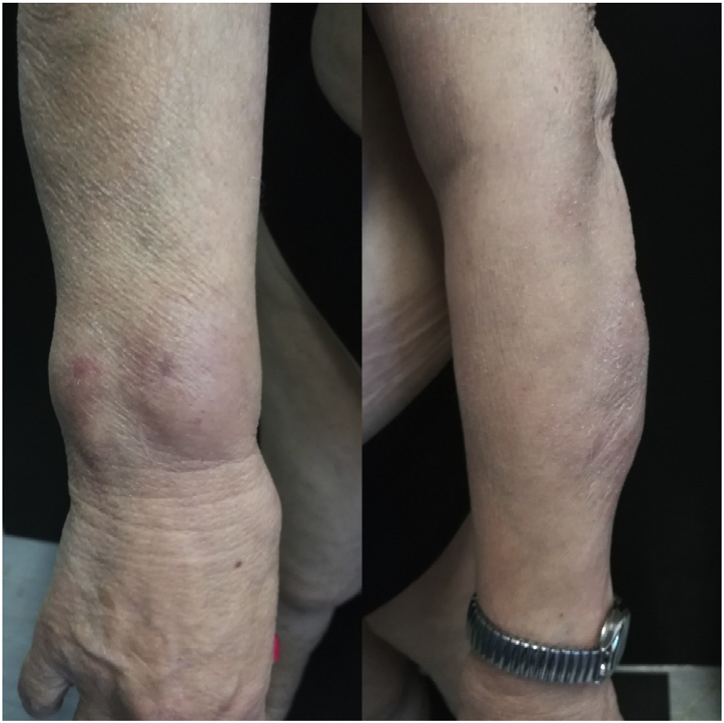
Sarcoid-like lesions. Multiple erythematous-violaceous indurated nodules bilaterally distributed on the arms of a 73-year-old woman.

**Fig 2:**
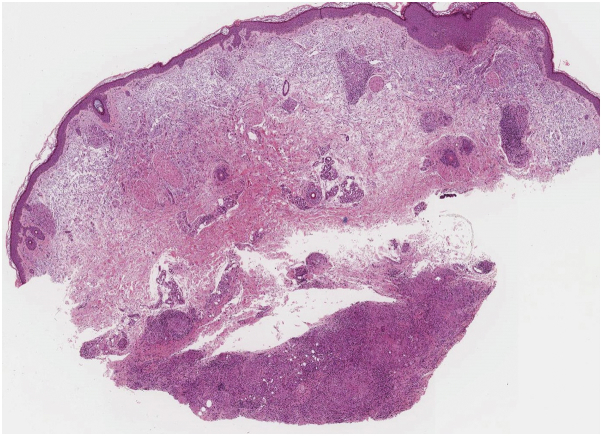
Sarcoid-like lesions. Biopsy performed on the right forearm showing intact epidermis, elastosis of the reticular dermis, and nonnecrotizing giant-cell granulomatous reaction predominantly affecting the hypodermis (Hematoxylin-eosin stain; original magnification: ×10.)

Sarcoidosis is a multisystem disease, the pathogenesis of which remains unclear. The condition is characterized by noncaseating granulomas developing in various organs, in genetically predisposed individuals. The induction of a granulomatous reaction is related to challenge with immunogenic stimuli such as antigens from mycobacteria, rickettsia, and viruses.

Stimulation with pathogen-associated molecular patterns, such as viral RNA-triggered pattern-recognition receptor signaling, results in dendritic cell and macrophage activation. These cells release type-1 proinflammatory mediators (interleukin 12), priming CD4^+^ T lymphocytes toward a T-helper 1 (Th1) cell-mediated response. The disease immune response is characterized by oligoclonal expansion of CD4^+^ T lymphocytes and production of Th1-proinflammatory cytokines, such as interleukin 2, tumor necrosis factor alfa, and interferon gamma. These further enhance macrophage activation in a “vicious circle” pattern that ultimately results in epithelioid giant-cell-granuloma formation.^3^^,^^4^ BNT162b2 encodes the RBD of the SARS-CoV-2 spike protein, eliciting an RBD-specific response with CD8^+^ and CD4^+^ T-cell expansion, interferon gamma release, and strong antibody production.^5^

We hypothesize that SARS-CoV-2 mRNA was the first trigger in the development of a cutaneous sarcoid granulomatous reaction. A subsequent challenge with the nucleoside-modified SARS-CoV-2 mRNA in the vaccine may have caused a disease relapse through activation of macrophages and dendritic cells, stimulating RBD-specific memory Th1 cells. Since this localized manifestation is not an absolute contraindication to completion of the vaccination cycle, we will consider administration of additional doses after remission of inflammatory lesions, recommending low-dose systemic immunosuppressants to prevent relapse.

Our report highlights the risk of reactivation of COVID-19-related lesions following SARS-COV-2 vaccination.

## Conflicts of interest

None disclosed.
